# Taste and Smell Disorders in Children and Young Adults With Cystic Fibrosis and Primary Ciliary Dyskinesia—A Prospective Comparative Study

**DOI:** 10.1002/ppul.71419

**Published:** 2025-12-05

**Authors:** Lea Christiane Beermann, Lisa Sophie Demski, Lynn Eitner, Stefanie Dillenhöfer, Anne Schlegtendal, Claire Mallon, Folke Brinkmann, Christoph Maier, Thomas Lücke, Anna Teresa Hoffmann

**Affiliations:** ^1^ University Children's Hospital Ruhr University Bochum Bochum Germany; ^2^ Division of Paediatric Pneumology & Allergology University Medical Center Schleswig‐Holstein Luebeck Schleswig‐Holstein Germany; ^3^ Member of the German Center for Lung Research (DZL) Airway Research Center North ARCN Germany; ^4^ Division of Rare Diseases and Center for Rare Diseases Ruhr, Member of the European Reference Network on Rare Endocrine Conditions University Children´s Hospital, Ruhr‐University Bochum Bochum Germany

**Keywords:** cystic fibrosis transmembrane conductance regulator, hypogeusia, hyposmia, smell disorders, taste disorders

## Abstract

**Background:**

In cystic fibrosis (CF), the defect of the CF transmembrane conductance regulator (CFTR) can also affect sensory nerve cell function, as recently demonstrated in animal models. The aim of this prospective cohort study was to investigate whether taste and smell disorders in CF correlate with persistent CFTR dysfunction detectable by iontophoresis or rather with inflammation or lung function. Participants with primary ciliary dyskinesia (PCD) and controls without pulmonary disease served as comparators.

**Methods:**

In 65 participants (age median 19 years IQR [12−26]; CF *n* = 23, PCD *n* = 22, controls *n* = 20) at the University Children´s Hospital Bochum, we measured taste (salty, sweet, sour, bitter) at four concentrations (“Taste‐Strips,” score 0−16, hypogeusia age‐adjusted < 8/< 9/< 9.9/< 10 points) and smell (“U‐Sniff”‐test, score 0−12, reduced odor identification performance < 8 points), pilocarpine iontophoresis, spirometry, inflammatory markers (e.g., CRP) and subjective chemosensory impairment. Statistics: Chi²/Fisher's‐exact, Mann−Whitney‐*U*, Kruskal−Wallis, linear regression; *p* < 0.05.

**Results:**

Hypogeusia occurred only in CF (17.4%). Particularly misidentification of the taste “salty” occurred significantly more frequently in CF (34.8% vs. PCD 19.3% and controls 17.5%), especially in the CF subgroup with elevated sweat chloride ≥ 60 mmol/l. Reduced odor identification performance was significantly more common in PCD (30% vs. CF 4%). Chemosensory disorders were not related to current lung function or inflammation.

**Conclusion:**

Taste disorders in CF are mostly attributed to difficulties tasting salty and are associated with elevated sweat chloride, probably caused by increased salivary salt following CFTR dysfunction in salivary glands rather than in the nerve cells. Smell disorders, however, remain a significant issue, particularly in PCD.

**Abbreviations**
CF: cystic fibrosis
CFTR: cystic fibrosis transmembrane conductance regulator
CRP: C‐reactive protein
ENT: ear‐nose‐throat
FEV_1_
: forced expiratory volume in the first second
IL: interleukin
IQR: interquartile range
PCD: primary ciliary dyskinesia
pwCF: people with cystic fibrosis
pwPCD: people with primary ciliary dyskinesia
TDI: threshold, discrimination and identification
TNF: tumor necrosis factor

## 1 Introduction

Cystic fibrosis (CF) is a rare autosomal‐recessive disorder caused by CFTR (CF transmembrane conductance regulator) chloride channel gene mutation with disrupted chloride secretion [1]. This leads to thickened mucus, impaired mucociliary clearance, and chronic rhinosinusitis with nasal polyposis [1]. Data from animal models demonstrate that CFTR channel dysfunction also impairs neuronal cell function, leading to reduced axon density and decreased nerve conduction velocity [2]. Recently, CFTR has been detected in human brains and cranial nerves [3]. Whether CFTR dysfunction correlates with chemosensory disorders in people with CF (pwCF) is unknown. Smell dysfunction has been reported in 40%−80% of adults [4, 5, 6, 7], and olfactory threshold and discrimination were impaired in children with CF [8]. Research on gustatory function in chronic pulmonary diseases remains limited. Older studies in pwCF often focused exclusively on salt taste [9, 10] and showed contradictory results. There is only one previous study in adult pwCF performing standardized testing (“Taste Strips”) including all four taste qualities [5]. However, detecting chemosensory dysfunction is crucial, as it can impact safety [11], mental health, and food intake [11]. Particularly in CF, it may compromise nutrition and aggravate weight loss due to malabsorption [4].

Similar to pwCF, olfactory dysfunction was observed in up to 70% of people with PCD (pwPCD) [12, 13, 14]. PCD is an autosomal‐recessive or x‐linked motile ciliopathy with impaired mucociliary clearance, also resulting in frequent upper airway infections and chronic sinonasal inflammation [12, 15]. In pwPCD, gustatory function has only been assessed once using electrogustometry [16], not showing taste disorders.

Notable is the discrepancy between subjective impairment and test‐based olfactory disorders in both CF [4] and PCD [16]. Previous authors hypothesized a lack of awareness due to the early onset of upper airway involvement in childhood [4].

The pathophysiological mechanisms of chemosensory disorders in chronic pulmonary diseases may be manifold. Besides CFTR dysfunction in CF, chronic inflammation is a possible cause for smell [17, 18] and taste dysfunction [19] in CF and PCD. Furthermore, pulmonary function as a marker of disease severity has been analyzed for its correlation with chemosensory disorders in CF [5, 20], but, to our knowledge, not in PCD.

The aim of this study was to quantify the frequency of taste and smell disorders in pwCF and pwPCD in comparison with each other and age‐matched controls without pulmonary disease, and to identify possible associations with CFTR dysfunction, decreased pulmonary function, or chronic inflammation.

## 2 Materials and Methods

### 2.1 Study Design and Participants

We conducted this monocentric prospective study as part of a larger project on pain and neural complications in CF and PCD at the University Children's Hospital Bochum, Germany. Additionally, the study was promoted by the PCD patient organization “Kartagener Syndrom und Primäre Ciliäre Dyskinesie e.V.”. The overall study was performed in accordance with the Declaration of Helsinki, approved by the ethical committee of the Ruhr University Bochum in March 2022 (Reg. No.: 22‐7467) and registered in the German Clinical Trials Register (DRKS‐Nr.00030406). We obtained written informed consent (in children verbal consent).

From November 2022 to April 2024, we recruited 65 participants: 23 with CF, 22 with PCD, and 20 age‐matched controls without known lung disease (Supporting Information S1: 1). Inclusion criteria for CF were a confirmed diagnosis according to the CF Foundation Consensus Guidelines [21], for PCD a confirmed diagnosis or at least “highly likely” according to the European Respiratory Society guidelines criteria [15] and for all an age over 7 years. Missing consent, language barrier, acute infection with fever, acute pulmonary exacerbation [22] ≤4 weeks, acute SARS‐CoV‐2 infection ≤ 3 months, and Long‐COVID syndrome were exclusion criteria.

The tests were performed by two examiners in randomized order to reduce a bias of inter‐observer variability. According to study protocol, all examinations had to be completed within ≤4 weeks. Except for one child, for whom we had to perform the taste test on another day due to inattention, all participants completed all tests on 1 day.

### 2.2 Gustatory and Olfactory Testing

For taste assessment, we used “Taste Strips” (Burghardt Messtechnik GmbH, Holm, Germany) according to the standardized testing protocol [23] validated from age five [23, 24]. The test consists of 18 filter paper strips, two tasteless and 16 impregnated with the qualities sweet, sour, salty and bitter in four concentrations [23]. After an hour of fasting, participants had to taste the strips and identify the taste from a set of four pictures. One point was given for each flavored strip identified correctly (max. 16 points) and quantity and quality of parageusia (wrong identification) were recorded. The cut‐off < 9 points in participants older than 15 years (in those ≤ 15 years age‐adjusted: < 8 points for boys 8−9 years/< 10 points for girls 8−9 years/< 10 points for boys 10−15 years/< 9.9 points for girls 10−15 years [24]) indicated hypogeusia according to the 10th percentile in healthy participants [23, 24]. Moderate hypogeusia was defined 9−12 points (age‐adjusted: 8−12/9.9−12/10−12 points), reflecting a decline of more than one standard deviation from the mean taste score in our control group (13.65 ± 1.66) following a new proposed classification (Mallon et al. I can taste it, but it's disgusting. Submitted 04/25, currently in revision). Furthermore, we defined the inability to identify salt in the lowest or second‐lowest concentration as salt taste disorder.

For olfactory testing, we used the “U‐Sniff”‐test (Burghardt Messtechnik GmbH, Holm, Germany), validated from age 6‐17 [25, 26]. Participants had to smell 12 felt‐tip pens (“Sniffin' Sticks”) with well‐known odors (e.g., banana, fish, rose) according to the standardized protocol [25] and choose from four pictures (one target, three distractors). Participants received one point for each smell identified correctly (max. 12 points). As the test only measures odor identification, and a concurrent assessment of odor threshold and discrimination is recommended [27] for participants over 17 years old to diagnose “hyposmia,” we have decided to use the term “reduced odor identification performance” to accurately reflect the findings of olfactory testing of this study group. The cut‐off value for reduced odor identification performance was <8 points [25, 28] and for moderate reduced odor identification performance 8−10 points in alignment with the classification of moderate hyposmia proposed by Lukasik et al. [29].

Subjective impairment was evaluated by interview, assessing occurrence and impact on daily life.

### 2.3 Other Data

Participants were asked about their medication, current symptoms, prior medical history (respiratory tract infections, allergies, otorhinolaryngological surgeries, exacerbations within the last 6 months), and comorbidities. The data were completed by the responsible physician at our department. Clinical examination included lung auscultation and anterior rhinoscopy with a nasal speculum to recognize large polyps Meltzer Grade 4 [30].

Sweat chloride concentration was measured with quantitative pilocarpine iontophoresis [31] using the Macroduct Advanced System and ChloroChek Chloridometer (ELITechGroup Inc., Logan, Utah, United States). A concentration of ≥ 60mmol/L was considered elevated and indicative of relevant CFTR dysfunction, consistent with the diagnostic cut‐off for CF [31].

Spirometric measurement was carried out by experienced personnel of our clinic. We used FEV_1_ (forced expiratory volume in the first second) with a cut‐off of 80%—equivalent to a *z* value below −1.96—to define reduced pulmonary function.

To assess systemic inflammation, we quantified serum inflammatory markers. C‐reactive protein (CRP), interleukin‐1β (IL‐1β), interleukin‐6 (IL‐6), and tumor necrosis factor α (TNF‐α) were measured in collaboration with laboratory at University Hospital Charité, Berlin, using LEGENDplex Human Essential Immune Response Panel by BioLegend. We used the established normative values applied by our laboratory (CRP < 5 mg/L; IL‐1β < 16 pg/mL; IL‐6 < 7 pg/mL; TNF‐α < 12.2 pg/mL [32]).

### 2.4 Statistical Analysis

Based on olfactory data in CF [4, 5, 8], sample size calculation using G*Power (power 95%, *α* error level 0.05) indicated a minimum of 14 participants per group. The calculated sample sizes for the overall project were larger, and all participants underwent the full set of measurements. We performed statistical analysis with SPSS 29.0.2.0 (IBM Corporation) using Shapiro−Wilk test, Chi‐square test, Fisher's‐exact, Kruskal−Wallis, *t*‐test, Mann−Whitney‐*U* test and univariate linear regression analysis. Differences were considered statistically significant at *p* ≤ 0.05.

## 3 Results

### 3.1 Baseline Characteristics

There were no relevant group differences regarding gender, age, history of allergy (Table 1), and further clinical characteristics (Table 2). FEV_1_ was on average 10% lower in participants with PCD than in CF (Table 2), but the difference was not significant. Only the frequency of previous Ear Nose and Throat surgery (ENT surgery; mainly polypectomy/adenoidectomy) was with three quarters in PCD significantly higher than in CF and controls (Table 1). Extensive nasal polyposis (Meltzer grade 4) was found in three participants by nasal inspection (PCD *n* = 2, CF *n* = 1). No participant had an acute exacerbation (exclusion criterion), yet about 30% of CF and 60% of PCD presented with pathological lung auscultation. None of the participants took any medication known to affect smell or taste (e.g., Linezolid). 83% of CF had at least one copy of F508del, the most common CFTR mutation in CF [1]. In PCD, the most common mutation was DNAH5 with 27% (details on genetics in Supporting Information S1: 2).

**Table 1:** Demographic and clinical data of participants with cystic fibrosis, primary ciliary dyskinesia and controls.

	Cystic fibrosis	Primary ciliary dyskinesia	Controls	*p* value			
				Primary ciliary dyskinesia versus cystic fibrosis	Cystic fibrosis versus controls	Primary ciliary dyskinesia versus controls	All three groups
*N*	23	22	20				
Females	11 (47.8%)	15 (68.2%)	13 (65%)	*p* = 0.231	*p* = 0.359	*p* = 1.000	
Age in years (median, IQR)	18 (10.5…24)	18 (12.3…29.8)	22,5 (13…25.5)				*p* = 0.665
Age groups							*p* = 0.867
8−12 years	8 (34.8%)	6 (27.3%)	4 (20%)				
13−18 years	4 (17.4%)	5 (22.7%)	5 (25%)				
>18 years	11 (47.8%)	11 (50%)	11 (55%)				
Previous ENT surgery^a^ (*n* = 64^b^)							
any ENT surgery	7 (30.4%)	16 (76.2%)	4 (20%)	***p*** = **0.003**	*p* = 0.501	***p*** < **0.001**	
previous polypectomy/adenoidectomy	7 (30.4%)	13 (61.9%)	0 (0%)	*p* = 0.068	—	—	
History of allergy (*n* = 61^b^)							
at least one	11 (47.8%)	8 (42.1%)	10 (52.6%)	*p* = 0.763	p = 1.000	*p* = 0.746	

*Note:* Percentages in brackets refer to the total number of available data in this category (missing answers excluded). Bold values are statistically significant.

Abbreviation: ENT = ear, nose and throat.

^a^ Polyposis surgery, adenoid surgery, tympanostomy tube, tonsillectomy;

b Number of answers available in this category.

**Table 2:** Clinical data of participants with cystic fibrosis and primary ciliary dyskinesia.

	Cystic fibrosis *n* = 23	Primary ciliary dyskinesia *n* = 22	*p* value
Current medication			
Antibiotics (long term)	3 (13%)	1 (4.5%)	*p* = 0.608
Steroids (oral or inhalative)	5 (21.7%)	8 (36.4%)	*p* = 0.337
Pancreatic enzymes	22 (95.7%)	—	—
Inhalation therapy^a^	21 (91.3%)	19 (86.4%)	*p* = 0.665
Other	16 (69.6%)	5 (21.7%)	*p* = 0.090
Not any medication	0 (0%)	9 (39.1%)	—
Current modulator therapy			
Kaftrio	16 (69.6%)	—	
Orkambi	1 (4.3%)	—	
None	6 (26.1%)	—	
Disease exacerbation within last 6 months			
At least one	8 (34.8%)	6 (60%)	*p* = 0.257
Missing	0	12	
Lung function			
Median FEV_1_ in % (IQR)	95 (82.5…105.5)	84 (69…95)	*p* = 0.061
FEV_1_ ≤ 80%	4 (17.4%)	6 (50%)	*p* = 0.059
Missing	0	10	

*Note:* Percentages in brackets refer to the total number of available data in this category (missing answers excluded).

Abbreviation: FEV_1_ = Forced expiratory volume in the first second.

a Inhalation therapy: hypertonic saline, salbutamol, recombinant human deoxyribonuclease I, or tiotropium bromide (excluding inhalative steroids).

### 3.2 Taste

**Figure 1:**
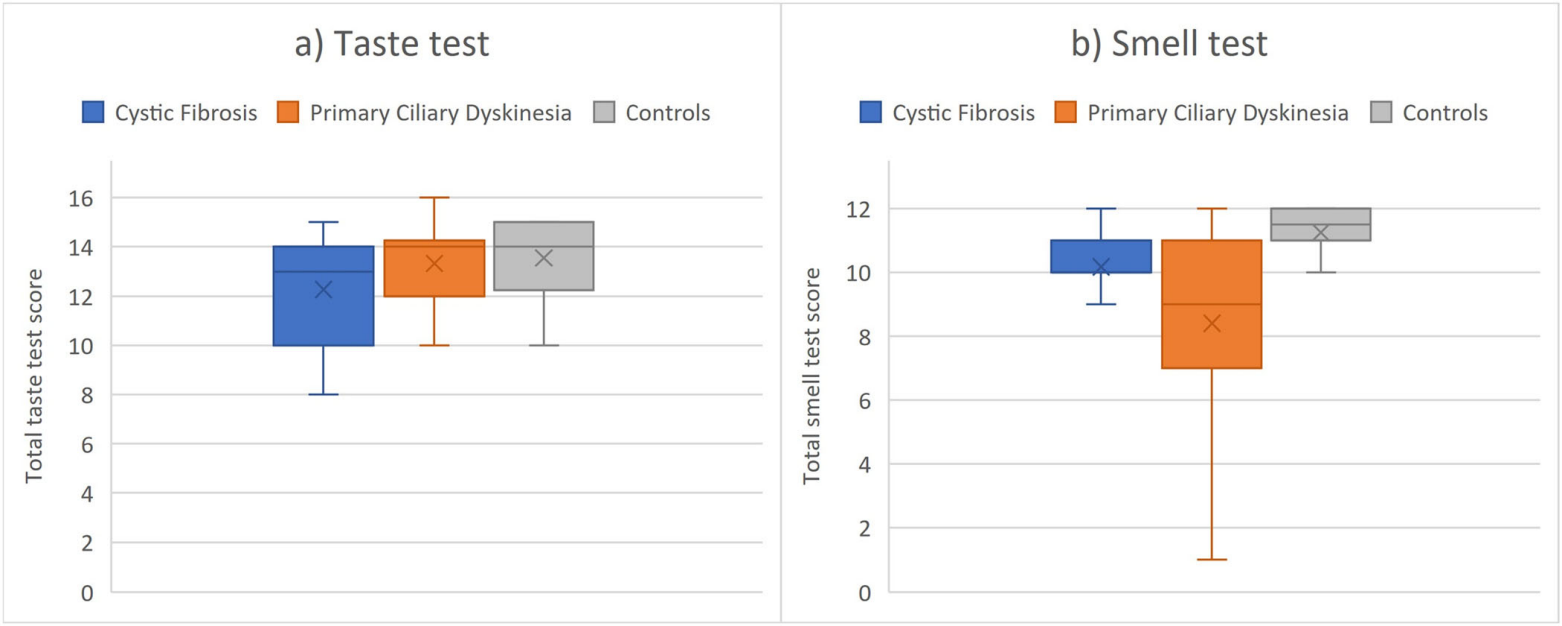
Distribution of taste and smell test scores in cystic fibrosis, primary ciliary dyskinesia and controls. (a) Distribution of total taste test score values. (b) Distribution of total smell test score values. The “x” in the figure indicates the mean values of taste and smell test scores in each group. Number of participants per group: cystic fibrosis *n* = 23; primary ciliary dyskinesia *n* = 22; Controls *n* = 20. [Color figure can be viewed at wileyonlinelibrary.com]

**Figure 2:**
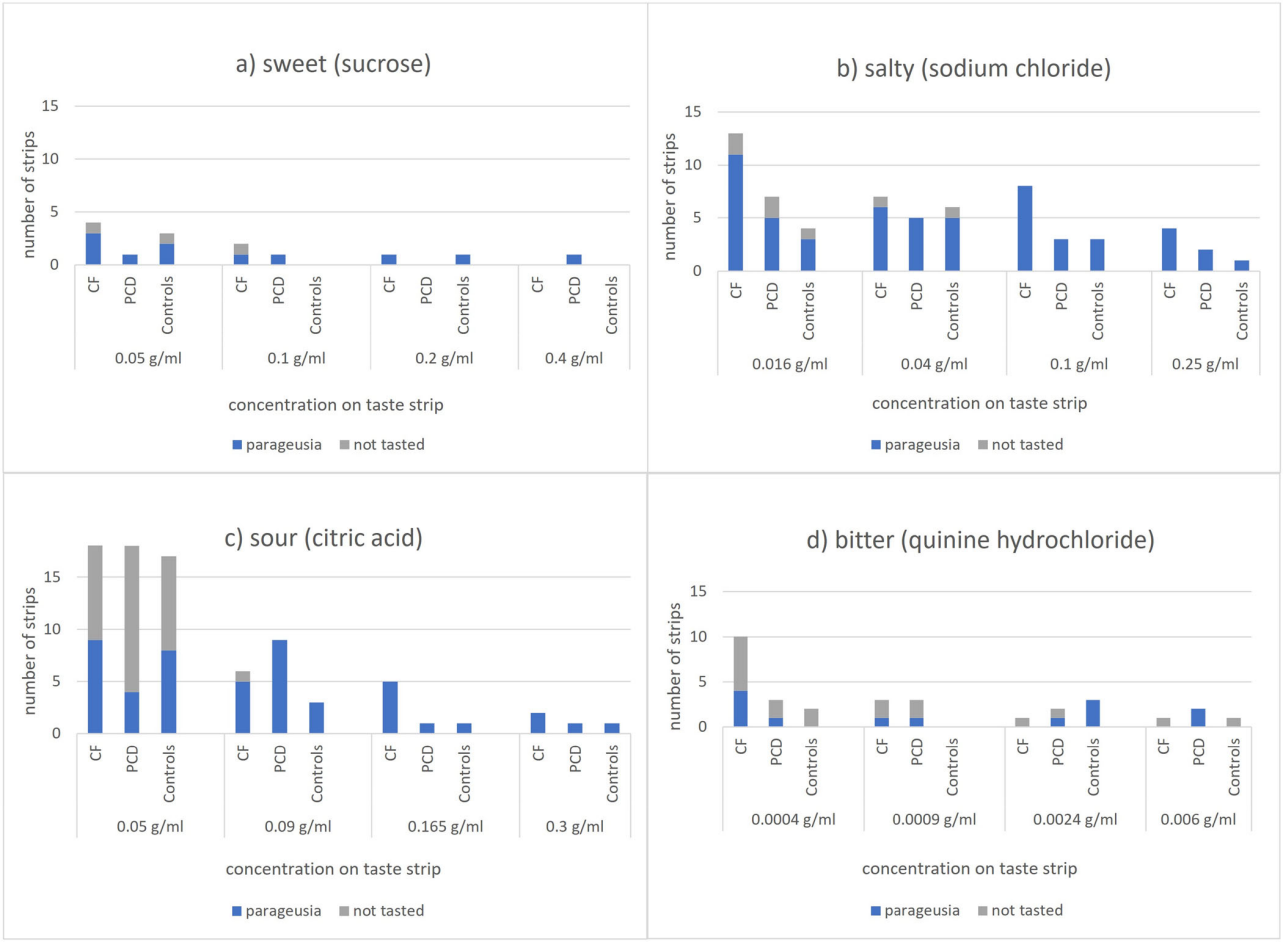
Number of wrong and not tasted strips per group (cystic fibrosis, primary ciliary dyskinesia and controls) according to taste quality (sweet, salty, sour, bitter) and four different concentrations within each quality. (a) Sweet taste strips (sucrose), (b) Salty taste strips (sodium chloride), (c) Sour taste strips (citric acid) and (d) Bitter taste strips (quinine hydrochloride). Parageusia = wrong taste identification; PCD = primary ciliary dyskinesia; CF = cystic fibrosis. [Color figure can be viewed at wileyonlinelibrary.com]

Hypogeusia (cut‐off age‐adjusted <8/<9/<9.9/<10 points) only occurred in CF (Table 3). One‐third of participants with PCD exhibited moderate hypogeusia (age‐adjusted 8−12/9−12/9.9−12/10−12‐points age‐adjusted), whereas a quarter of CF and controls did (Table 3, Figure 1). In both CF (*R*² = 0.005, *p* = 0.760) and PCD (*R*² = 0.000, *p* = 0.989), there was no significant influence of age as an independent variable on the total taste test score in a univariate linear regression model. The amount of wrong and not‐tasted strips across the four different concentrations of each taste quality (dose‐response pattern) was similar in all three groups, except for salty. Participants with CF misidentified salty (parageusia and ageusia, 34.8%) significantly more often than PCD (19.3%; *p* = 0.029) and controls (17.5%; *p* = 0.015) (Figure 2). All CF participants with abnormal taste test (≤12‐points) showed a salt taste disorder (inability to identify salt in the lowest or second‐lowest concentration), whereas only half of those with PCD did (*p* = 0.023). The sum of incorrect identifications for sweet, sour, and bitter (excluding errors on salty strips) did not differ significantly between the groups: CF misidentified 19.6% of the strips, PCD 15.9% and controls 13.8%.

**Table 3:** Results of taste and smell test and subjective impairment in all groups.

	Cystic fibrosis	Primary ciliary dyskinesia	Controls	*p* value	
				Cystic fibrosis versus primary ciliary dyskinesia	All three groups
Taste test					
*n*	23	22	20		
Median score [IQR]	13 (10.5…14)	14 (12…14)	14 (12.8…15)	*p* = 0.217	*p* = 0.191
Minimum−maximum score	8−15	10−16	10−16		
Hypogeusia	4 (17.4%)	0 (0%)	0 (0%)	*p* = 0.109	***p*** = **0.020**
Moderate hypogeusia	6 (26.1%)	8 (36.4%)	5 (25%)	*p* = 0.530	*p* = 0.662
Normogeusia	13 (56.5%)	14 (63.6%)	15 (75%)	*p* = 0.763	*p* = 0.447
Subjective taste impairment*	0 (0%)	3 (14.3%)	0 (0%)	*p* = 0.100	***p*** = **0.043**
Smell test					
*n*	23	20	20		
Median score [IQR]	10 (10…11)	9 (7…11)	11.5 (11…12)	*p* = 0.143	***p*** = **0.005**
Minimum−maximum score	0−12	1−12	9−12		
Reduced odor identification performance	1 (4.3%)	6 (30%)	0 (0%)	***p*** = **0.038**	***p*** = **0.005**
Moderate reduced odor identification performance	11 (47.8%)	5 (25%)	3 (15%)	*p* = 0.206	*p* = 0.054
Normosmia	11 (47.8%)	9 (45%)	17 (85%)	*p* = 1.000	***p*** = **0.015**
Subjective smell impairment^b^	2 (8.7%)	9 (47.4%)	0 (0%)	***p*** = **0.011**	***P*** < **0.001**

*Note:* Cut‐off values: hypogeusia age‐adjusted < 8/<9/<9.9/<10 points; moderate hypogeusia age‐adjusted 8−12/9−12/9.9−12/10−12‐points; reduced odor identification performance < 8 points; moderate reduced odor identification performance 8−10 points. Percentages in brackets refer to the total number of available data in this category (missing answers excluded). Bold values are statistically significant.

Abbreviation: IQR = interquartile range.

a Data available in *n* = 21 participants with primary ciliary dyskinesia;

b Data available in *n* = 19 participants with primary ciliary dyskinesia.

**Figure 3:**
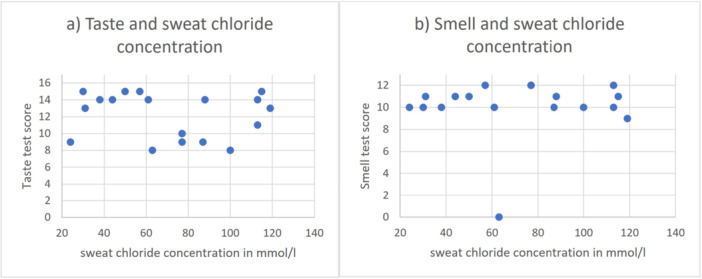
Association of total taste and smell test scores with sweat chloride concentration in pilocarpine iontophoresis in participants with cystic fibrosis. (a) Association of total taste test score values and sweat chloride concentration. (b) Association of total smell test score values and sweat chloride concentration. Data available in *n* = 20 participants with cystic fibrosis. A concentration of ≥ 60 mmol/L chloride in sweat was considered elevated and indicative of relevant CFTR dysfunction. Elevated sweat chloride concentrations ≥ 60 mmol/L occurred in *n* = 11 (55%) of participants with CF. In CF with ≥ 60 mmol/L sweat chloride, total taste test scores were lower, with the difference being near significance (*p* = 0.067, Mann−Whitney‐*U* test). The distribution of total smell test scores across the subgroups ≥ 60 mmol/l and < 60 mmol/L did not differ (*p* = 0.941, Mann−Whitney‐*U* test). [Color figure can be viewed at wileyonlinelibrary.com]

In CF with elevated sweat chloride ≥ 60 mmol/L (*n* = 11/20; Figure 3), abnormal taste tests occurred more than twice as often (54.4% vs. 22.2% in CF with < 60 mmol/L; *p* = 0.197) and total taste scores were lower (*p* = 0.067). There was a significant increase in salt taste disorders (90.9% vs. 22.2%; *p* = 0.005) and in misidentifications of salty (*p* = 0.020) in the ≥ 60 mmol/L group compared to CF with < 60 mmol/L sweat chloride.

FEV_1_ was decreased (≤ 80%) in less than a quarter of CF and in half of PCD (Table 2). The severity of pulmonary involvement appears to be unrelated to taste disorders in both CF and PCD, as participants with abnormal taste tests (Supporting Information S1: 3) as well as salt disorders in CF (63.2% vs. 75.0%) were similarly distributed across the subgroups FEV_1_ > 80% and ≤ 80%.

At least one inflammatory marker (CRP, IL‐1β, IL‐6, or TNF‐α) was elevated in about 40% of CF and two thirds of PCD (IL‐1 β Supporting Information S1: 3, other markers Supporting Information S1: 4). Frequency of abnormal taste tests didn't differ significantly between the subgroups of inflammatory status (at least one inflammatory marker elevated vs. all in normal range) in CF (55.5% vs. 41.6%) and PCD (40% vs. 40%). Also, salt taste disorders occurred similarly often in CF with elevated and normal inflammatory markers (55.5% vs. 66.6%).

Subjective taste impairment (Table 3) was only reported in PCD (*n* = 3), although test‐based hypogeusia was not present in PCD but in 9% of CF.

### 3.3 Smell

Nearly one‐third of PCD presented with reduced odor identification performance (cut‐off < 8 points), whereas only one participant with CF and none of the controls did (Table 3, Figure 1). Univariate linear regression analysis did not show any significant influence of age on the total smell test score, neither in CF (*R*² = 0.097, *p* = 0.147) nor in PCD (*R*² = 0.006, *p* = 0.745).

All participants with reduced odor identification performance reported a history of ENT surgery. There was no significant difference in the frequency of moderate reduced odor identification performance (8−10 points) between CF and PCD, but the prevalence was almost significantly lower in the control group (Table 3). Olfactory dysfunction was present in all three participants with Meltzer grade 4 polyposis, one exhibiting reduced odor identification performance and two moderate reduced odor identification performance.

There was no association between sweat chloride concentration (Figure 3), FEV_1_ value, or blood level of inflammatory markers and smell test score, neither in CF nor in PCD (Supporting Information S1: 3, Supporting Information S1: 4).

Subjective smell impairment was significantly more common among PCD than CF (Table 3). All participants with test‐based reduced odor identification performance also reported subjective impairment, but few participants (CF *n* = 1, PCD *n* = 3) reported subjective impairment, whereas no objective disorder was measurable.

## 4 Discussion

In summary, a third of PCD and a quarter of CF had moderate taste disorders, whereas severe taste disorder (hypogeusia) was only present in CF. An isolated salt taste disorder occurred significantly more frequently in CF. Remarkably, this salt taste disorder was four times more common among CF with elevated sweat chloride. Smell disorders occurred in both CF and PCD, but severe disorders appeared almost exclusively in PCD with one‐third of participants being hyposmic. There was no association between chemosensory disorders with chronic inflammation or reduced pulmonary function.

The rare frequency of hypogeusia in CF we found is consistent with the few previous studies. Two recent studies in pwCF aged 5 years and older also comprehensively assessing all four taste qualities [5, 20] didn't find any differences compared to healthy controls. Earlier studies showed contradictory results: One found an increased gustatory sensitivity in CF [33], which could never be confirmed [9, 10]. Some of these studies with small groups of CF (*n* ≤ 11) observed phenomena of reduced salt sensitivity similar to our findings [9, 10], although not significant compared to controls. Our data suggest an association of taste disorders with elevated sweat chloride, indicating a pathophysiological relation to persistent CFTR dysfunction. In animal models, CFTR dysfunction was associated with impaired nerve function. But because the taste disorders in CF were predominantly based on reduced salt perception and the increase in misidentifications of other taste qualities in CF was only slight and not significant, a general negative influence of CFTR dysfunction on neuronal taste perception cannot be substantiated from our data. A more convincing answer is provided by studies detecting significantly elevated salivary concentrations of sodium chloride in CF mice [34] and pwCF [35]. Furthermore, adaption to increased salivary sodium chloride was associated with reduced salt sensitivity [36]. In this context, the repeated therapeutic application of hypertonic saline in CF should also be considered. Based on our data and that of another study using electrogustometry [16], there is no indication that hypogeusia plays any role in PCD. Moderate hypogeusia was found in a third of PCD and in a quarter of CF and controls. This cut‐off is relatively new (Mallon et al. I can taste it, but it's disgusting. Submitted 04/25, currently in revision), prior data in CF and PCD are not available. Evaluating pathophysiological backgrounds of gustatory disorders, we analyzed associations with pulmonary function and systemic inflammation. In agreement with other authors [5, 20], decreased FEV_1_ values were not associated with gustatory dysfunction in CF. Prior data in PCD are not available. Interestingly, taste dysfunction and inflammation also were not associated, which contrasts with reports on inflammation‐related gustatory deficits in other medical conditions [17, 18, 19].

Reduced odor identification performance occurred only in 4% of participants with CF, whereas previous studies report 40%−80% hyposmia in adults [4, 5, 6, 7, 37] and impaired olfactory threshold and discrimination in children by TDI test (Threshold, Discrimination and Identification) [8]. The low prevalence we observed can be explained in part using the “U‐Sniff”‐test also in adults, although the test is specifically designed for children and only validated for the age of 6−17 years. This is particularly important, as research on normative values for the TDI test revealed a disproportionately high prevalence of hyposmia in young participants [38]. At the same time, one study using the age‐adjusted “U‐Sniff”‐test found 40% hyposmia in children with CF [39]. This indicates, the extrapolation to young adults in this study could have led to an underestimation of olfactory deficits, as sensory threshold and discrimination are not covered by the “U‐Sniff”‐test. In CF, especially olfactory threshold may be impaired following chronic rhinosinusitis with or without nasal polyposis [4]. A recent study reported, that 10% of their participants (children and adults with CF) were normosmic on identification but impaired in the threshold test [27]. Although the univariate linear regression analysis did not reveal any significant influence of age on the smell test scores in our participants, the conclusions regarding the prevalence of olfactory dysfunction in CF remain limited in this study and are primarily representative in the context of comparison with PCD. It should also be considered that nearly three‐quarters of participants with CF in this study received modulator therapy, known to reduce nasal polyposis [40] associated with smell dysfunction [18]. In PCD, our finding of 30% reduced odor identification performance is as well below that of prior studies (74% by TDI test [12] and 52% by “U‐Sniff”‐test [16]). In the latter study [16], the authors state that most participants did not receive adequate rhinosinusitis treatment [16]. Participants in this study might experience less severe upper airway involvement, receiving specific sinusitis treatment (nasal steroids or nasal saline sprays/irrigation) in nearly 40%. All participants with reduced odor identification performance reported a history of ENT surgery. Whether there is a causal relation between ENT surgery and smell disorders or whether the need for ENT surgery indicates severe upper airway involvement is unclear. Also, possible functional deficits of olfactory cilia in PCD as motile ciliopathy have been discussed [12] but not conclusively assessed. Moderate reduced odor identification performance was significantly less common in the control group than in PCD and CF. The only study including assessment of mild/moderate hyposmia in PCD [13] found 72% smell disorders, whereas we observed reduced odor identification performance in 55%. Their participants' median age was 20 years older, and the authors themselves demonstrated reduced smell with increasing age. Decreased FEV_1_ was not associated with lower smell test scores, consistent with three previous studies in CF [5, 20, 27]. Prior data in PCD are not available. Inflammation and olfactory disorders were not associated in CF and PCD, neither of which, to our knowledge, has been studied. The differences in prevalence of subjective impairment and test‐based disorders observed in CF and PCD have been described before [4, 16], emphasizing the need for objective testing.

The key strength of this study lies in using a taste test with a comprehensive assessment of all four qualities, allowing us to detect the specific salt taste disorder in CF, whereas other qualities are mostly unaffected. Confounding effects of age‐related decline in olfaction [13, 14, 18] and gustation [19] on comparisons between groups can be excluded given the homogeneous age structure across all groups.

## 5 Limitations

Due to the monocentric design and CF and PCD being rare diseases, our findings are limited by the sample size. The “U‐Sniff”‐test is only validated for 6−17 years, therefore, the extrapolation to young adults in this study allows only limited conclusions regarding the prevalence of olfactory disorders in CF and PCD and the findings primarily provide information in the context of comparison with each other. Furthermore, there is a risk of selection bias by possibly recruiting pwPCD and pwCF, particularly interested in healthcare and treatment. Lung function and inflammatory markers could not be assessed in all participants because some refused the blood draw or were unable to perform spirometry for organizational reasons. Two controls for whom a therapy with Lisdexamfetamine was identified after testing were not excluded secondarily, as they did not show any current signs of attention deficit hyperactivity disorder. Although likely minimal, the impact of this medication cannot definitively be eliminated. Lastly, we used psychophysical chemosensory tests, which may be influenced by reduced concentration/motivation.

## 6 Conclusion and Implications

This study shows taste disorders in CF, which are mainly attributed to reduced salt sensitivity. They are associated with elevated sweat chloride levels, indicating persistent CFTR dysfunction. A general negative effect of CFTR dysfunction on neuronal perception of smell and taste cannot be substantiated from our data. Adaptation phenomena to increased salivary salt content seem more plausible and require further investigation. Olfactory disorders however, remain a widespread condition, particularly in PCD. Our data suggest no association of pulmonary function or inflammation and chemosensory disorders, but further research in larger study populations is required.

## Author Contributions

**Lea Christiane Beermann:** investigation, writing −,original draft, visualization, formal analysis, validation, data curation, writing − review and editing. **Lisa Sophie Demski:** investigation, writing − review and editing; data curation. **Lynn Eitner:** methodology, resources, writing − review and editing, supervision. **Stefanie Dillenhöfer:** resources, investigation, writing − review and editing. **Anne Schlegtendal:** investigation, resources, writing − review and editing. **Claire Mallon:** writing − review and editing, methodology, resources. **Folke Brinkmann:** conceptualization, writing − review and editing. **Christoph Maier:** conceptualization, methodology, writing − review and editing, data curation, supervision. **Thomas Lücke:** conceptualization, writing − review and editing, funding acquisition. **Anna Teresa Hoffmann:** project administration, conceptualization, investigation, funding acquisition, writing − review and editing, supervision.

## Ethics Statement

Approved by the local ethics committee of the Ruhr University Bochum (Reg. No.: 22‐7467, 09.03.2022) and registered in the German Clinical Trials Register (DRKS‐Nr.00030406).

## Conflicts of Interest

The authors declare no conflicts of interest.

## Supporting information


**Supporting 1:** STROBE flowchart. **Supporting 2:** Distribution of mutations in participants with cystic fibrosis and primary ciliary dyskinesia. **Supporting 3:** Association of total taste and smell test scores with pulmonary function (FEV_1_) and serum concentration of the inflammatory cytokine IL‐1β. **Supporting 4:** Detailed data on taste and smell test results in association with serum inflammatory cytokines IL‐6, IL‐1β, TNFα and CRP.

## Data Availability

The data that support the findings of this study are available from the corresponding author upon reasonable request.
