# Supplementary material for: Taste and Smell Disorders in Children and Young Adults With Cystic Fibrosis and Primary Ciliary Dyskinesia—A Prospective Comparative Study
Source: Pediatr Pulmonol. 2025 Dec 5;60(12):e71419. doi: 10.1002/ppul.71419 (PMC12679337; doi:10.1002/ppul.71419)
Supplement: Supplementary file 1 — Supporting 1: STROBE flowchart. Supporting 2: Distribution of mutations in participants with cystic fibrosis and primary ciliary dyskinesia. Supporting 3: Association of total taste and smell test scores with pulmonary function (FEV1) and serum concentration of the inflammatory cytokine IL‐1β. Supporting 4: Detailed data on taste and smell test results in association with serum inflammatory cytokines IL‐6, IL‐1β, TNFα and CRP. [file PPUL-60-0-s001.rtf]

Supplements

Supplement 1: STROBE flowchart.
Overview of the recruitment process for participants with Cystic Fibrosis, Primary Ciliary Dyskinesia and controls.
CF = Cystic Fibrosis; PCD = Primary Ciliary Dyskinesia.


Supplement 2: Distribution of mutations in participants with Cystic Fibrosis and Primary Ciliary Dyskinesia.
* heterozygote and homozygote (n=9) F508del mutation
** other mutations in Primary Ciliary Dyskinesia: CCDC40, DNAAF1, DYX1C1, DNAI1, CFAP300, c.29092delT, 
c.3021G>A.
Cystic Fibrosis
n=23	Primary Ciliary Dyskinesia
n=22	
F508del*	19 (82.6%)	DNAH5	6 (27.3%)	
N1303K	2 (8.7%)	DNAH11	2 (9.1%)	
G542X	1 (4.3%)	RSPH4A	2 (9.1%)	
c.3425_3587del	1 (4.3%)	other**	6 (27.3%)	
		no information available	6 (27.3%)	


Supplement 3: Association of total taste and smell test scores with pulmonary function (FEV1) and serum concentration of the inflammatory cytokine IL-1â.
Data available in each category: FEV1 value (Cystic Fibrosis: n=23; Primary Ciliary Dyskinesia: n=12), IL-1â (Cystic Fibrosis: n=21, Primary Ciliary Dyskinesia: n=15). FEV1= forced expiratory volume in the first second; IL-1â = interleukin 1â.


Supplement 4: Detailed data on taste and smell test results in association with serum inflammatory 
cytokines IL-6, IL-1â, TNFá and CRP.
IL-6 = interleukin 6; IL-1â = interleukin 1â; TNFá = tumour necrosis factor alpha; CRP = C-reactive protein. 
Normative values: CRP <5mg/l; IL-1â <16 pg/ml; IL-6 <7 pg/ml; TNFá <12.2 pg/ml.
Cut-off values: hypogeusia age-adjusted <8/<9/9.9/<10 points; moderate hypogeusia age-adjusted 8-12/9-12/9.9-12/10-12 points; reduced odor identification performance <8 points; moderate reduced odor identification performance 8-10 points. Percentages in brackets refer to the total number of available data in this subgroup.

Inflammatory markers (CRP, IL-1â, IL-6 and TNFá)	
all normal	at least one elevated	
	Cystic Fibrosis	n=12	n=9	
hypogeusia	3 (20%)	1 (11.1%)	
moderate hypogeusia	2 (16.6%)	4 (44.4%)	
salt taste disorder	8 (66.6%)	5 (55.5%)	
reduced odor identification performance	1 (8.3%)	0 (0%)	
moderate reduced odor identification performance	6 (50%)	4 (44.4%)	
	Primary Ciliary Dyskinesia	n=5	n=10	
moderate hypogeusia	2 (40%)	4 (40%)	
reduced odor identification performance	1 (20%)	4 (40%)	
moderate reduced odor identification performance	2 (40%)	2 (20%)	

IL-6	
normal	
elevated	
	Cystic Fibrosis	n=15	n=6	
		hypogeusia	3 (20%)	1 (16.7%)	
		moderate hypogeusia	4 (26.7%)	2 (33.3%)	
		reduced odor identification performance	1 (6.7%)	0 (0%)	
		moderate reduced odor identification performance	8 (53.3%)	2 (33.3%)	
	Primary Ciliary Dyskinesia	n=8	n=7	
		moderate hypogeusia	2 (25%)	4 (57.1%)	
		reduced odor identification performance	2 (25%)	3 (42.9%)	
		moderate reduced odor identification performance	3 (37.5%)	1 (14.3%)	

IL-1â	
normal	
elevated	
 	Cystic Fibrosis	n=18	n=3	
 		hypogeusia	4 (22.2%)	0 (0%)	
 		moderate hypogeusia	5 (27.8%)	1 (33.3%)	
 		reduced odor identification performance	1 (5.6%)	0 (0%)	
 		moderate reduced odor identification performance	10 (55.6%)	0 (0%)	
 	Primary Ciliary Dyskinesia	n=10	n=5	
 		moderate hypogeusia	3 (30%)	3 (60%)	
 		reduced odor identification performance	2 (20%)	3 (60%)	
 	 	moderate reduced odor identification performance	4 (40%)	0 (0%)	

TNFá	
normal	
elevated	
	Cystic Fibrosis	n=20	n=1	
		hypogeusia	4 (20%)	0 (0%)	
		moderate hypogeusia	6 (30%)	0 (0%)	
		reduced odor identification performance	1 (5%)	0 (0%)	
		moderate reduced odor identification performance	10 (50%)	0 (0%)	
	Primary Ciliary Dyskinesia	n=12	n=3	
		moderate hypogeusia	4 (33.3%)	2 (66.7%)	
		reduced odor identification performance	3 (25%)	2 (66.7%)	
		moderate reduced odor identification performance	4 (33.3%)	0 (0%)	

CRP 	
normal	
elevated	
	Cystic Fibrosis	n=20	n=3	
		hypogeusia	4 (20%)	0 (0%)	
		moderate hypogeusia	4 (20%)	2 (66.7%)	
		reduced odor identification performance	1 (5%)	0 (0%)	
		moderate reduced odor identification performance	9 (45%)	2 (66.7%)	
	Primary Ciliary Dyskinesia	n=10	n=5	
		moderate hypogeusia	6 (60%)	0 (0%)	
		reduced odor identification performance	3 (30%)	2 (40%)	
		moderate reduced odor identification performance	3 (30%)	1 (20%)	
